# Effects of a Supervised-As-Needed Home Exercise Program on Scoliosis and Motor Function in Rett Syndrome: A Multiple-Baseline Study

**DOI:** 10.3390/jcm14061873

**Published:** 2025-03-11

**Authors:** Alberto Romano, Marina Luisa Rodocanachi Roidi, Miriam Nella Savini, Ilaria Viganò, Michal Dziubak, Luca Pietrogrande, Daniel Sender Moran, Meir Lotan

**Affiliations:** 1 Department of Health System Management, Ariel University, Ariel 4070000, Israel; 2 Developmental Age Neurology, Epilepsy Center, San Paolo Hospital, 20142 Milan, Italy; 3 Orthopedic and Traumatology, San Paolo Hospital, 20142 Milan, Italy; 4 Department of Health Sciences, University of Milan, 20142 Milan, Italy; 5 Department of Physiotherapy, Ariel University, Ariel 4070000, Israel; 6 Israeli Rett Syndrome National Evaluation Team, Ramat Gan 5200100, Israel

**Keywords:** Rett syndrome, scoliosis, motor skills, telerehabilitation, physical therapy modalities, home exercise program

## Abstract

**Background/Objectives:** Scoliosis is a prevalent comorbidity in Rett syndrome (RTT), often necessitating surgical intervention. This study investigated the impact of a 10-month individualized home exercise program (HEP) on scoliosis progression and gross motor function in girls aged six to 16 years with RTT. **Methods:** A multiple-baseline single-case design (AABA) was employed with 20 participants. A remotely supervised HEP, based on established principles focused on posture and physical activity, was implemented daily for at least one hour. The primary outcome was the rate of scoliosis progression assessed through the Cobb angle change measured via spinal radiographs at baseline, pre-intervention, and post-intervention. The secondary outcome was the gross motor function. **Results:** The HEP did not significantly reduce the rate of scoliosis progression. However, individual responses varied, with three participants showing scoliosis reduction. Significant improvements were observed in gross motor function, particularly in standing, walking, and stair-climbing abilities. **Conclusions:** The HEP did not significantly impact overall scoliosis progression, but a significant improvement was found in gross motor function. Further research into larger sample sizes is needed to confirm the effectiveness of exercise interventions in people with RTT.

## 1. Introduction

Rett syndrome (RTT) is a severe neurodevelopmental disorder primarily affecting females, with an estimated prevalence of 5 to 10 in 100,000 female births [1]. RTT is caused by MECP2 gene mutations, which encode a protein critical for brain development [2,3,4]. The clinical history of people with RTT is characterized by a seemingly typical development followed by a regression of acquired skills between 6 and 18 months of age [5,6]. The regression manifests as a loss of communication skills, the development of stereotypical hand movements, and impairments in gross motor function [7,8].

Neurogenic scoliosis is a prevalent comorbidity in RTT, affecting about 75% of individuals [9,10]. It contributes to various complications, including pain, impaired sitting balance, diminished motor skills, and the potential for restrictive lung disease [11,12]. Scoliosis in RTT typically progresses rapidly, with an average increase of 14° to 21° Cobb per year [13,14,15,16], and this progression often accelerates during puberty [10,17,18,19]. The reported median age of scoliosis onset is 9.8 years [20], but scoliosis in RTT can emerge earlier than four years of age [17,18], with 25% of cases emerging by 6 years of age, 79% by 13 years, and 85% by 16 years or older [10,20]. Factors such as specific genetic mutations, early motor development delay, poorer walking and sitting skills, the onset of puberty, and increased clinical severity are associated with an earlier onset of scoliosis and worsened scoliosis progression [17,18,21,22].

Although conservative management, including bracing and physiotherapy, may be employed initially, spinal fusion surgery is often necessary for curves exceeding 40–50° Cobb [10]. Even if surgery can improve spinal alignment, pain, and respiratory function [23,24], it presents significant challenges for the child and the child’s family due to the complexity of the procedure and the inherent risks associated with surgical intervention in this population [25].

Despite the high prevalence and significant impact of scoliosis in RTT, there remains a critical need for effective conservative management strategies, especially for individuals who may not be suitable candidates for surgery or for whom surgery is delayed. Conservative treatments of scoliosis are critical during the onset of puberty and adolescent growth spurt, identified as a crucial period for scoliosis progression [18,26].

Physical activity interventions have shown promise in improving motor function and quality of life in individuals with RTT [27,28]. However, the efficacy of such interventions in mitigating scoliosis progression has not been extensively studied. Lotan and colleagues [29] introduced a conservative physical therapy intervention comprising an intensive program of postural positioning and enhanced physical activity level carried out in close cooperation with the participant’s family members in a single case study. The intervention demonstrated a reduction of 10° Cobb in the scoliosis in a 5-year-old girl with RTT over 18 months of treatment. Recently, Romano et al. [30] proposed an intervention involving therapeutic activities for parents at home with their daughters, easily integrated into daily routines. The activities included passive (hypercorrective), active postural strategies (activated through balance reactions), and motor tasks to strengthen trunk muscles. Twenty participants with RTT were evaluated before and after the intervention. The median Cobb’s angle progression was 2.1° (range: −13–23°), and three subjects improved their curve for at least 10° Cobb. Two were 3.8 and 6.5 years, respectively, and the third was 31.6 years. Although encouraging, these studies lack control groups, and the results do not focus on the phase of puberty where scoliosis was reported to progress the most in RTT. Therefore, the protective effect of a treatment employing physical activity and postural strategies for reducing scoliosis progression of people with RTT in that critical phase is still to be proved.

This study aims to evaluate the effect of a ten-month individualized home exercise program (HEP) for delayed [29,30] progression of scoliosis in a cohort of children and adolescents with RTT aged between six and 16 years. The research hypothesis for this study is that the scoliosis progression rate reduces when people with RTT are included in an individualized HEP designed explicitly for scoliosis progression reduction.

## 2. Materials and Methods

This study employs a multiple-baseline single-case design (AABA) across a series of participants. Letters “A” represent the evaluation session, while the letter “B” represents the intervention phase. The independent variable was implementing a 10-month individualized motor and postural activity program carried out by participants’ parents and care providers within the participants’ daily living environment for at least one hour a day, five days a week.

Ethical approval was obtained from the Ariel University Institutional Review Board (AU-HEA-ML-20201019) and Milan Area 1 Ethical Committee (protocol no. 0034568/2023). The study was conducted following the Helsinki Declaration and registered in a public trial registry (ClinicalTrial.gov, ID: NCT05488938, approval date: 3 August 2022). No specific checklist is established for multiple-baseline single-case designs in physical rehabilitation. The closest existing checklist is the Single-Case Reporting Guideline In Behavioral Interventions (SCRIBE) checklist. However, it is primarily designed for studies focused on behavioral interventions and does not contain the elements to adequately report a study evaluating a motor rehabilitation intervention for scoliosis. The study adheres to rigorous reporting standards.

### 2.1. Participants

A sample size of 20 participants was determined through a power analysis with an alpha of 0.05 and a desired power of 0.80 to be sufficient to detect a clinically meaningful difference of 5° Cobb in scoliosis progression between the baseline and intervention phases. Based on previous studies, this calculation considered the anticipated effect size (ES) and the variability in scoliosis progression observed in individuals with RTT [13,14,15]. Eligibility criteria included a confirmed diagnosis of RTT with a MECP2 mutation, diagnosis of scoliosis (Cobb angle 10–40°) measured within six months before the eligibility assessment, and age between 6 and 16 years. Exclusion criteria were neurodevelopmental deficits within the first six months of life, a history of neurometabolic disease, infections, or perinatal brain injury, unstable health conditions (e.g., recurring infections, severe gastrointestinal issues, or uncontrolled epilepsy) that, in the evaluating physician’s judgment, precluded participation, prior or planned spinal surgery during the study period, and the use of a daytime back brace. Upon recruitment, participants’ legal kin received an information sheet about the study and provided informed consent for participation and video material collection.

### 2.2. Procedure

Candidates’ eligibility was assessed through a neurological or psychiatric evaluation. After inclusion confirmation, the baseline (T0) spine radiograph was collected for each participant. After 10 months (T1), all participants underwent a second spine radiograph and a comprehensive rehabilitation assessment. No changes were applied to the participants’ rehabilitation routine between T0 and T1. Pre-intervention assessment (T1) was conducted by a team of specialists, including a physiotherapist and a neuro-psycho-motor therapist with at least five years of experience in RTT rehabilitation and a rehabilitation technician experienced in constructing and building postural assistive devices for individuals with complex physical disabilities. This assessment aimed at (i) creating an individualized intervention plan tailored to the participant’s scoliosis and functional abilities, (ii) gathering baseline and outcome measures, and (iii) identifying each participant’s postural needs to guide the orthopedic technician in constructing or adjusting assistive devices such as wheelchairs, posture support systems, standing aids, and adapted chairs.

Following the T1 assessment, an individualized HEP focusing on posture and activity was developed for each participant. Based on the participants and their families’ daily schedules, the HEPs were designed to be implemented daily for over 10 months at their educational facilities and homes. Each HEP incorporated activities tailored to the participant’s needs and abilities, leading to considerable variation between programs. However, all programs adhered to core rehabilitation strategies, which included (i) activities and exercises performed in sitting, standing, and walking positions that challenged the participant’s balance reactions and encouraged asymmetrical muscle activation to oppose the scoliosis curve, (ii) asymmetrical postures and passive stretching (hypercorrective positioning) to elongate shortened trunk muscles, and (iii) spinal mobilization exercises, as supported by previous research [29,30].

**Figure 1:**
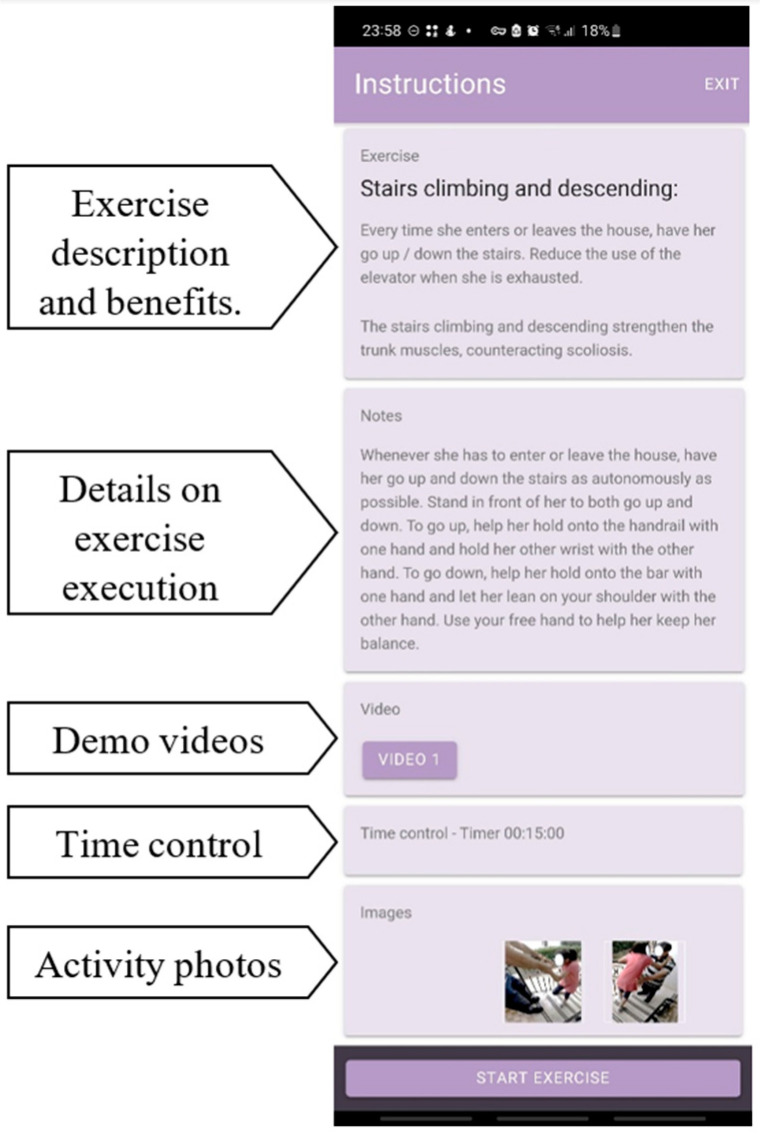
Screenshot from the “RettActive” application interface, designed to guide caregivers through various exercises. At the top, the Exercise field provides the name of the activity, a brief description of how to perform it, and an explanation of its benefits. Below this, the Notes field offers more detailed instructions and tips for successful execution. The app also includes a Video field, where caregivers can access short videos demonstrating the activity, and an image field with photos illustrating key aspects. A Time control field allows caregivers to set a timer for the activity, and finally, a Start exercise button is used to begin recording the session.

Parents and caregivers carrying out the HEP received thorough training on program activities to ensure proper implementation. Each activity was carefully integrated into the participant’s daily routine, considering the participant’s and caregivers’ schedules. A smartphone application (named “ActiveRett”) was developed to assist with HEP implementation, providing individualized therapeutic instructions, reminders, and a platform for data collection. The application offered caregivers timely reminders before each activity and included detailed descriptions, pictures, and videos for every exercise (Figure 1). Caregivers were asked to use the application to report any challenges that led to not performing or completing the agreed-upon exercises. A researcher reviewed such reports weekly, providing remote supervision via video calls as needed (when the exercises were not performed following the original plan suggested before intervention initiation). These supervision meetings focused on discussing exercise performance, solving emerging problems, reorganizing schedules, adjusting exercises, evaluating goal achievement, and setting additional goals. Participants underwent a final spinal radiograph at the end of the 10-month program (T2), and outcome measures were collected again.

### 2.3. Measures

#### 2.3.1. RTT Severity Level

The Rett Assessment Rating Scale (RARS) was assessed at T0 and used as a descriptive measure to determine and present the severity of participants’ RTT symptoms [31]. This tool was validated for use with the Italian population with RTT, ensuring its accuracy and reliability in measuring the condition’s impact [31].

#### 2.3.2. Scoliosis Progression

**Figure 2:**
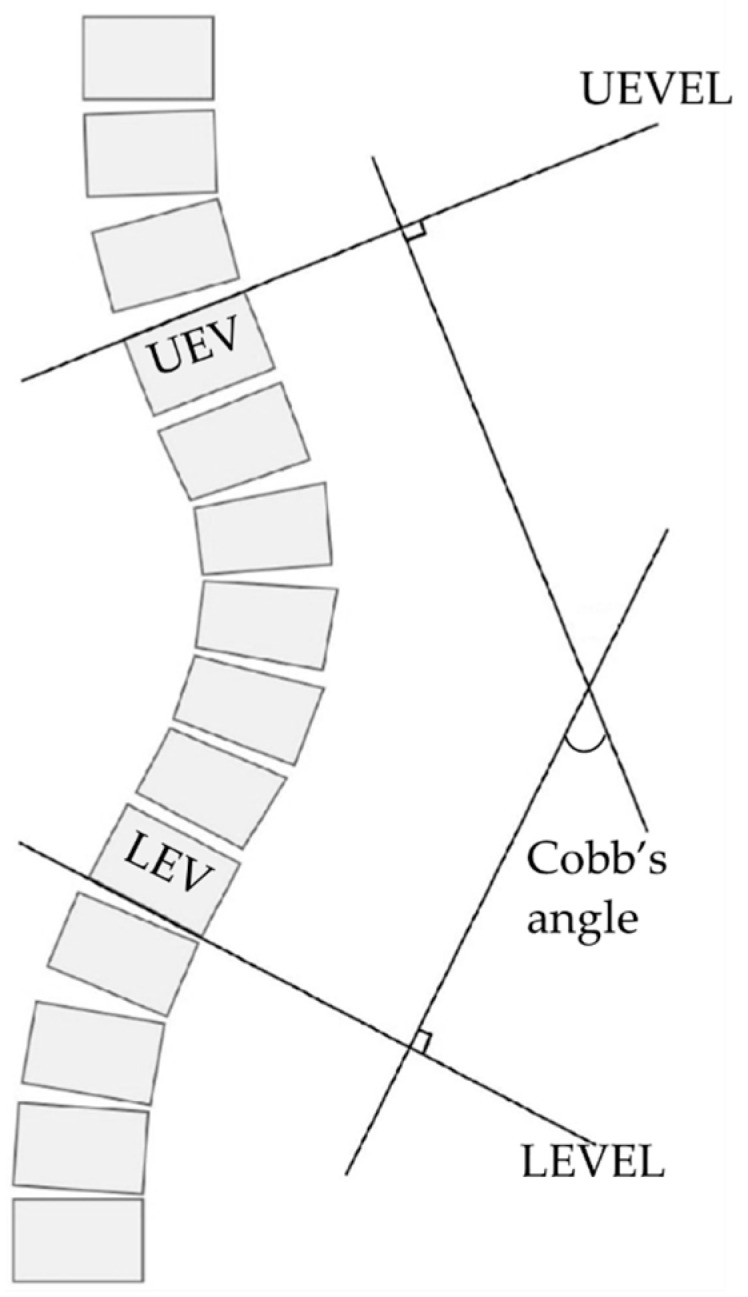
Visual explanation of the method used to measure the Cobb’s angle. Upper and lower end vertebra endplate lines (UEVEL; LEVEL). Reproduced with permission from Romano et al. [30].

The principal outcome measure was the progression of scoliosis before (T0–T1) and during the intervention (T1–T2). It was assessed by measuring the difference in Cobb’s angle from anteroposterior X-rays of the spine at T0, T1, and T2. A visual explanation of Cobb’s angle measurement procedure is available in Figure 2. Radiographs were collected for each participant at the same facility, in the same posture, and by the same technician, following international guidelines (Downs et al., 2009 [10]). The Cobb angle was determined by averaging the measurements of three independent, blinded medical specialists experienced with scoliosis in RTT. To ensure measurement blindness, the spinal radiographs were anonymized of any identifying information that could reveal the participants’ names or evaluation time points. Individual scoliosis improvement or worsening was intended as a change in Cobb’s angle higher or equal to 5° [32], which accounts for intra-observer and inter-observer variation.

#### 2.3.3. Gross Motor Function Level

As a secondary outcome measure, the participants’ gross motor function levels were evaluated before (T1) and after the treatment (T2) using the Rett Syndrome Motor Evaluation Scale (RESMES), an RTT-specific scale assessing the participant’s functional ability in standing, sitting, postural change, walking, running, and climbing/descending stairs [22,33]. Higher values represent the worst motor functioning. This scale showed strong internal consistency and optimal inter-rater agreement among clinicians [22].

### 2.4. Data Analysis

The dependent variables were Cobb’s angle change during the baseline (T0-T1) and intervention phases (T1–T2) and the change in gross motor functioning after the intervention (T1–T2). Due to the non-normal distribution observed in the data using the Shapiro–Wilk test, all statistical analyses were conducted using non-parametric methods. Cobb angle changes that occurred before (T0–T1) and during the treatment (T1–T2) were compared using the Wilcoxon signed-rank test. The same test was used to compare participants’ RESMES total and subscales scores at T1 and T2. The matched-pairs rank-biserial correlation calculated ES for statistically significant differences [34,35]. ES interpretation in this study adhered to the empirically derived guidelines established for rehabilitation research, wherein ES are categorized as small (0.140–0.310), medium (0.310–0.610), and large (>0.610) [36]. The raw Cobbs’ angles collected at T0, T1, and T2 were compared with Friedman’s test. Pairwise comparisons were conducted using the Durbin-Conover test. Potential relationships between changes in Cobb angles and age, baseline Cobb angle, RTT severity, baseline motor function, and changes in motor function were explored using Spearman’s rank correlation coefficient. The significance threshold for all comparisons was set at α = 0.05, with Bonferroni correction for multiple comparisons applied when needed [37].

## 3. Results

Twenty-two girls aged 6–16 years with genetically confirmed classic RTT and scoliosis were recruited from San Paolo Hospital (Milan, Italy) and two associations for people with RTT: the “Associazione Italiana Rett ETS” (Verona, Italy) and the Pro Rett Ricerca ONLUS association (Sermide e Felonica, Italy). Of these, 21 (95%) completed the 10-month intervention. One subject stopped the intervention due to severe health issues; another did not participate in the evaluation at T2 due to parental refusal to arrive at the final meeting and was excluded. Therefore, the final data analysis involved 20 subjects. Participants’ data are available as Supplementary Material (Table S1: individual participants’ data). Participants included in the analysis had a median age of 11.5 years (interquartile range–IQR: 4.1 years) and a median RTT severity measured with the RARS of 62 (IQR = 15.5) out of 124 at T0. Specifically, four subjects presented a mild RTT manifestation (RARS score <55) and one a severe manifestation (RARS score > 81). Moreover, the functional evaluation presented that all but two subjects were able to sit on a stool and the floor without support indefinitely; 10 participants (50%) were able to stand independently for more than one minute, and four (20%) could walk without support for more than 10 steps. At T0, enrolled participants performed a median amount of physical rehabilitative interventions of 120 min (IQR = 78.8 min).

### 3.1. Scoliosis Characteristics and Progression

Ten participants (50%) presented a C-shaped curve. Six of these were left-convex. In five cases, the C-shaped curves were large-radius scoliosis affecting the thoracolumbar area of the spinal column. In three cases, the curves were located in the lumbar area and, in two cases, in the dorsal tract. Ten participants (50%) showed an S-shaped curve. The primary curve was located in the dorsal area in seven of these subjects. The dorsal curves were right-convex in all participants. In three cases, the primary curve was left-convex and located in the lumbar area of the spinal column.

The median Cobb angle, considering all curves (primary and secondary), was 14.6° Cobb (IQR = 14.6° Cobb) at T0, 20.2° Cobb (IQR = 15.1° Cobb) at T1, and 27.0° Cobb (IQR = 19.8° Cobb) at T2. This change was statistically significant on Friedman’s test (*p* < 0.001). Pairwise comparisons revealed the statistical differences between T0 and T1 (*p* < 0.001) and between T1 and T2 (*p* = 0.025). Furthermore, the median yearly scoliosis progression rate was 6.0° (IQR = 7.0°) pre-intervention and 3.5° (IQR = 10.0°) post-intervention. The difference in progression rates before and after the intervention was not statistically significant (*p* = 0.657).

### 3.2. Individual Responses to Intervention

**Figure 3:**
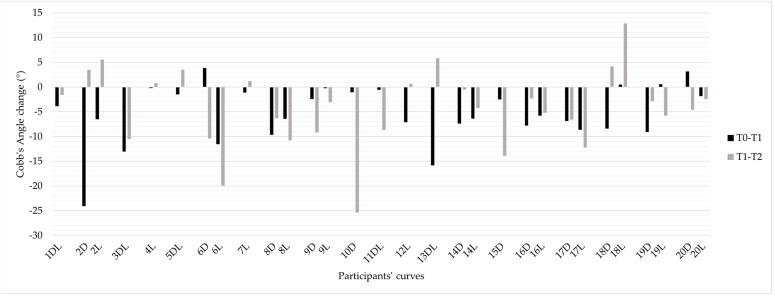
Cobb angle changes occurred during the baseline (T0–T1) and intervention (T1–T2) periods in each scoliotic curve of included participants. Positive values represent improvements (Cobb angle reduction), while negative values represent worsening (Cobb angle increment).

**Figure 4:**
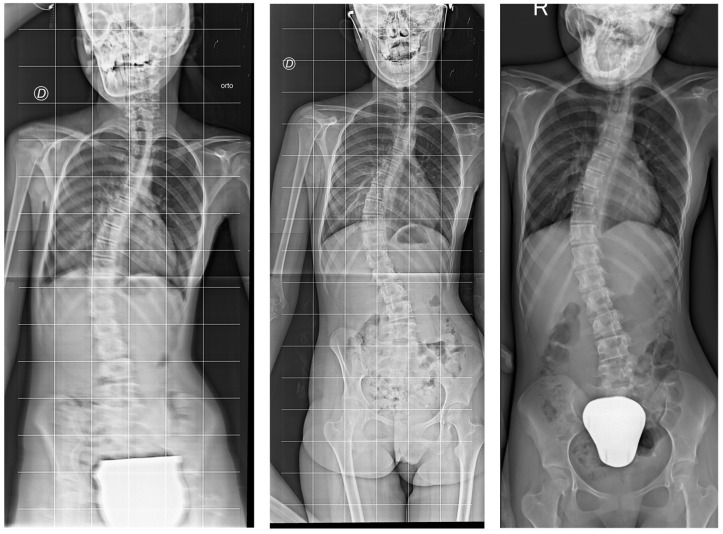
From (**left**) to (**right**), radiographs of participants no. 18 were collected at T0, T1, and T2, respectively. Letters “D” and “R” explicitate the right side of the body.

**Figure 5:**
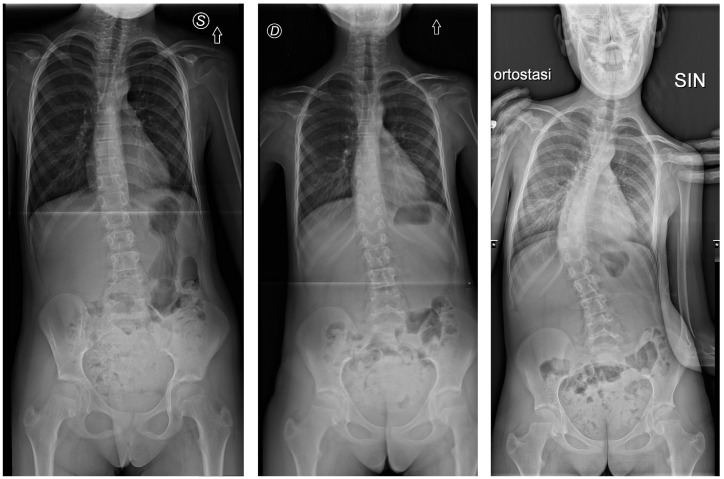
From (**left**) to (**right**), radiographs of participants no. 10 were collected at T0, T1, and T2, respectively. Letters “D” explicitates the right side of the body. The letter “S” and “SIN” indicate the left side of the body. The arrows indicate the upper margin of the radiographs.

Individual responses to the intervention varied. Figure 3 presents the participants’ Cobb angle change during the baseline and intervention. In the year preceding the intervention, 11 participants (55%) experienced a worsening of scoliosis (Cobb angle increase ≥ 5°), while nine (45%) remained stable. During the intervention, ten participants (50%) worsened their curve, seven (35%) remained stable, and three (15%) showed scoliosis improvement (Cobb angle reduction ≥ 5°). The radiographs of the participants who ameliorated and worsened the most during the intervention are depicted in Figure 4 and Figure 5, respectively.

Comparing individual changes in Cobb angle before and during the intervention revealed that six participants (30%) had a greater worsening of scoliosis during the intervention compared to the prior year. Conversely, seven participants (35%) showed less progression of scoliosis during the intervention than in the preceding year. In the remaining seven cases (35%), the change in Cobb angle was similar between the two periods.

### 3.3. Gross Motor Function

Before the intervention (T1), 15 participants (75%) could sit independently on a stool and the floor; 10 subjects (50%) were able to stand independently, while one (5%) could not stand even supported by the two arms; nine participants (45%) could walk unsupported for more than 10 steps and three (15%) were not able to perform any step when supported by the two arms.

**Figure 6:**
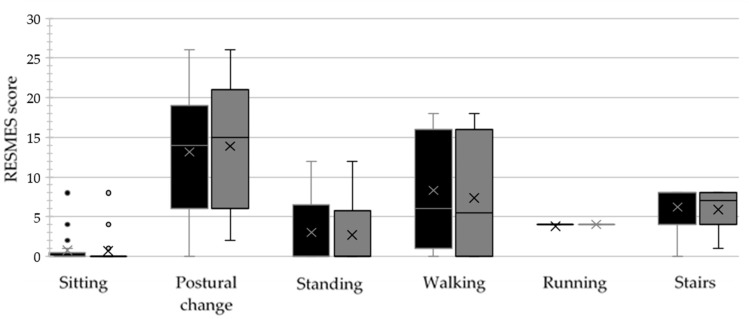
Participants’ scores in each RESMES subscale at T1 (black boxes) and T2 (gray boxes). The lower boundary of each box shows the 25th percentile, while the upper boundary shows the 75th percentile (excluding the median). The line and the cross within each box represent each distribution’s median and mean scores, respectively. The whiskers extend to the minimum and maximum values within the distribution, excluding outliers identified using Tukey’s method. Dots outside the boxes show distributions’ outliers.

The median RESMES total and subscale scores measured at T1 and T2 are collected in Table 1. Pre- and post-intervention data for each RESMES subscale are also depicted in Figure 6. Participants demonstrated significant improvement in gross motor function, as measured by the RESMES total (*p* = 0.002). No difference emerged in the RESMES subscales after correcting for multiple comparisons (α = 0.007). A total of 16 participants (80%) showed improvement, three (15%) remained stable, and one (5%) experienced a decline in motor function. The median improvement in RESMES scores was 2 points (IQR = 1).

Looking at the individual data, six subjects (30%) increased their standing ability, one of them (5%) learned to stand independently, and none worsened in this ability. Three participants (15%) ameliorated their maintenance of sitting position, while one (5%) worsened it. Six subjects (30%) improved in performing postural changes while worsening was observed in three cases (15%). Walking ability improved in nine (45%) participants; two of them (10%) became independent in this ability, and five (25%) learned to overcome at least one obstacle (3 cm high). One participant worsened her walking ability, requiring bilateral support instead of unilateral support. Finally, seven participants increased their ability to climb or descend a flight of stairs; two became independent in climbing a flight of stairs. One subject learned how to descend a flight of stairs with double support, but her climbing skill worsened, requiring double support instead of a single one.

## 4. Discussion

This study investigated the effect of a 10-month individualized HEP on scoliosis progression in a cohort of girls aged six to 16 with RTT. Contrary to the study hypothesis, the HEP did not significantly reduce the rate of scoliosis progression. However, it led to significant improvements in gross motor function, aligning with previous research [38,39].

### 4.1. Effect of the HEP on Scoliosis Progression

Although the median Cobb angle did not significantly increase during the intervention, no statistical difference existed between the progression rate during the baseline and intervention periods. This finding does not align with previous case studies that reported improving or stabilizing the Cobb angle following a similar intervention [29,30]. This discrepancy in the findings could be due to several factors. The present study included girls with RTT who face a critical age for scoliosis development in RTT [18,26], while previous investigations enrolled a wider range of ages, including older participants with stable scoliosis and functional abilities. Age is a critical factor for scoliosis progression. Girls and women before and after adolescence are less likely to experience a significant progression of the curve [10]. On top of age, four (20%) participants presented impressive growth spurts during the present investigation, which might have a negative effect on scoliosis development by increasing muscular imbalance [40]. Furthermore, the progression of scoliosis was monitored over a 10-month timeframe, whereas previous studies reported changes in Cobb angle following a shorter intervention [30]. In this previous research project, some participants attested to keeping the program “running” despite its difficulty, as they knew it would end in three months. A full-year program might be too lengthy. It might be better to separate such a demanding program into shorter sections, reducing care providers’ burden.

Moreover, the supervision of the HEP development was provided “as needed”, meaning that it was delivered when participants reported low adherence to the HEP or difficulties in its conduction. This approach leads to a median of seven supervision meetings (one every 1.4 months), while fortnightly supervision was provided in a similar previous study [29,30]. Supervision-as-needed offers greater flexibility for patients and clinicians and allows resources to be focused on cases that need more attention. However, it may not provide all individuals with the necessary level of support. Supervision-as-needed requires a strong patient-clinician trust relationship in which the patient feels confident in reporting the “negative” data that triggers the supervision delivery (e.g., low adherence or difficulties). This reporting bias (social desirability bias) affects the reliability of self-reported adherence [41], limiting access to supervision when a supervision-as-needed approach is used. On the other hand, a fixed-ratio supervision regimen ensures continuous monitoring of the person’s progress, allows timely adjustments to the program, and provides constant psychological and motivational support [42]. However, it requires significant time and resources from healthcare professionals and patients or families, and it may be challenging to sustain for a long time [27,43].

Therefore, from the current results, the proposed HEP appears insufficient to limit the progression of scoliosis in people with RTT aged between six and 16. Early intervention is highly recommended. Primary and secondary prevention strategies aimed at keeping the highest possible motor functioning, maintaining spine mobility, and balancing emerging spinal muscular asymmetries may enhance the effectiveness of HEP when scoliosis is diagnosed. Furthermore, the authors believe that a mixed supervision regimen, comprising periodic meetings and additional supervision when needed, may represent the best choice for remotely supervised HEP in people with RTT.

In addition to global results, individual responses to the intervention varied. While some participants experienced a worsening of scoliosis during the intervention, others showed a stabilization of the Cobb angle, and three subjects even improved the curve. This result, as well as those from Lotan et al. [29] and Romano et al. [30], represent the first report of scoliosis regression in RTT and are likely related to the implementation of HEP, as spontaneous scoliosis regression was only reported so far for this population when the HEP was implemented. However, the reasons underlying the fact that some participants ameliorated the scoliosis while others did not remain unclear and should be further investigated. This variability may be due to randomness and the interaction of several factors, including age, RTT symptoms severity, motor functioning, genetic mutation, and adherence to the HEP [17,18,20,44]. Moreover, it highlights the importance of implementing highly individualized and tailor-suited rehabilitation interventions for this population.

### 4.2. Effect of the HEP on Gross Motor Function

The HEP led to significant improvements in gross motor function, as measured by the RESMES. This finding is consistent with previous studies showing the beneficial effects of physical activity and HEP on motor function in individuals with RTT [28,45]. Motor functioning of people with RTT appears to improve following HEPs. This could be due to the change in daily routines accompanying the implementation of HEPs, which promote an active lifestyle and encourage primary caregivers to practice highly functional skills with the person with RTT regularly. The improvement observed may be attributed to several factors. The HEP likely provided opportunities to strengthen key muscle groups, improve balance and coordination, and increase overall physical activity levels. Furthermore, the individualized nature of the HEP ensured that the exercises were tailored to each participant’s specific needs and abilities, maximizing their potential for motor gains. Specifically, motor skills related to mobility, such as those related to standing, walking, and performing the stairs, are crucial to promote active movement and independence.

While our findings demonstrate significant motor improvements, the magnitude of improvement appears smaller than in previous studies focusing specifically on enhancing motor skills in RTT [38,45]. This could be due to the focus of the HEP. In the current research, the exercises were focused on reducing the progression of scoliosis instead of precisely enhancing motor skills. This speculation specifies the importance of focusing on specific aims when planning and providing HEPs to this population.

### 4.3. Limitations

This study has various limitations that should be considered. First, only twenty participants participated in the research, a small sample size that limits the findings’ external validity. RTT is a rare disorder that complicates the recruitment of participants and the development of large intervention studies. More extensive studies are recommended to confirm the current results and explore the factors linked to the variability in the outcomes. Furthermore, the multiple-baseline single-case design may not be as robust as a parallel-group design in detecting group differences. Third, the study did not include a control group. Therefore, although participants acted as controls for themselves (which stands to reason when considering the diverse clinical expressions presented by individuals with RTT), the observed effects could result from factors other than the HEP. In addition, no control period was implemented for motor functioning. Therefore, it is possible that the observed improvements in motor function were attributed to factors aside from the HEP. Furthermore, considering the rapid progression of scoliosis, a 10-month intervention may not suffice to observe significant changes in the scoliosis’s trajectory. Therefore, longer-term studies may be required to assess the true impact of the intervention. Moreover, no adherence measures were implemented. Self-reported adherence is prone to reporting biases, and further studies are needed to validate an objective adherence measure for remotely supervised HEPs [46]. 

## 5. Conclusions

This study provided preliminary information on the effect of an HEP on scoliosis progression and motor function in girls with RTT. While the HEP did not demonstrate a statistically significant reduction in the rate of scoliosis progression, significant improvements were observed in the gross motor functions total score. While the HEPs could be helpful in the overall management of individuals with RTT, they do not seem sufficient to slow down the rate of scoliosis progression when performed as a remote intervention. The lack of a control group and the absence of statistical significance in the primary outcome (scoliosis progression) underscore the need for further research. In particular, future studies, ideally randomized controlled trials with larger samples, should evaluate the effectiveness of different exercise interventions on scoliosis progression and motor function in individuals with RTT.

## Figures and Tables

**Table 1:** Median RESMES total and subscale scores were measured at T1 and T2. Higher values represent the worst motor functioning. ES was calculated if a significant difference between T1 and T2 (marked with an asterisk) was found.

	Subscale (Range)	Median (IQR)		*p*-Value (ES)
		T1	T2	
RESMES subscales	Sitting (0–12)	0 (0.25)	0 (0)	0.424
	Postural change (0–28)	14.5 (11.25)	15 (12.75)	0.669
	Standing (0–12)	0.5 (6.25)	0 (5.25)	0.026
	Walking (0–18)	8 (14)	5.5 (16)	0.009
	Running (0–4)	4 (0)	4 (0)	1.000
	Stairs (0–8)	8 (4)	7 (4)	0.021
RESMES total score (0–82)		33.5 (37)	31.5 (36.25)	0.002 * (0.850)

Abbreviation list: RESMES = Rett Syndrome Motor Evaluation Scale; IQR = Inter Quartile Range; ES = Effect Size.

## Data Availability

The original contributions presented in this study are included in the article/Supplementary Materials. Further inquiries can be directed at the corresponding author.

## Supplementary Materials

The following supporting information can be downloaded at https://www.mdpi.com/article/10.3390/jcm14061873/s1, Table S1: individual participants’ data.
